# Supplementary material for: CAncer bioMarker Prediction Pipeline (CAMPP)—A standardized framework for the analysis of quantitative biological data
Source: PLoS Comput Biol. 2020 Mar 16;16(3):e1007665. doi: 10.1371/journal.pcbi.1007665 (PMC7108742; doi:10.1371/journal.pcbi.1007665)
Supplement: S1 Fig — Plot showing the result of k-means clustering with k = 2. The two clusters support the presumed difference between N-glycan abundances in normal interstitial fluid vs tumor interstitial fluid samples. (PDF) [file pcbi.1007665.s001.pdf]

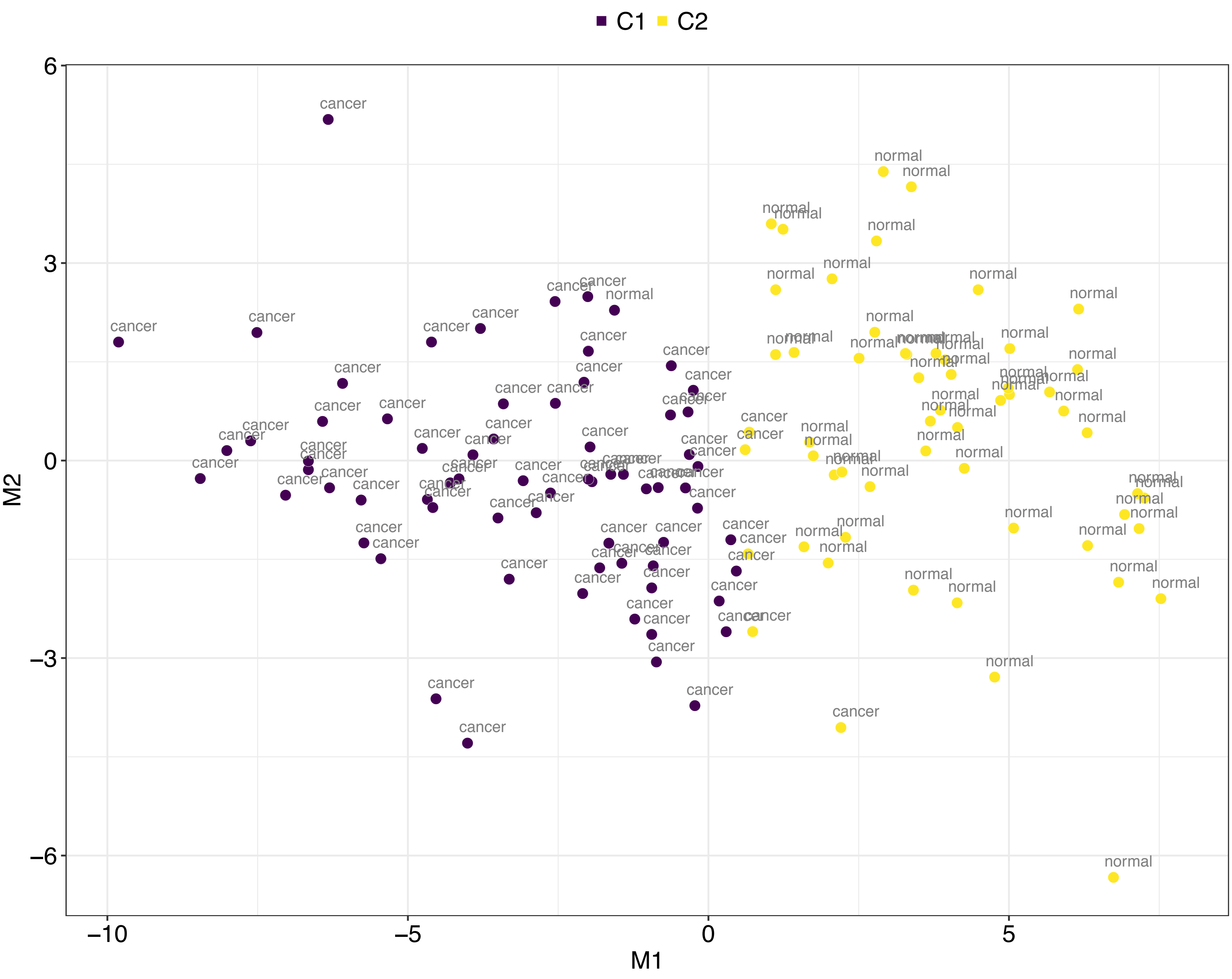

Supplementary Figure 1: Multidimensional scaling plot showing the result of k-means clustering with k = 2. The two clusters support the anticipated difference between N-glycan abundances in normal interstitial fluid vs tumour interstitial fluid samples.
