# Supplementary material for: CAncer bioMarker Prediction Pipeline (CAMPP)—A standardized framework for the analysis of quantitative biological data
Source: PLoS Comput Biol. 2020 Mar 16;16(3):e1007665. doi: 10.1371/journal.pcbi.1007665 (PMC7108742; doi:10.1371/journal.pcbi.1007665)
Supplement: Attachment — Submitted filename: ReplyPLOSComp.docx [file pcbi.1007665.s003.docx]

(1.)

* *the R code uses a lot of bioconductor packages but itself has an unusual install approach. why isn’t it packaged up as a bioconductor package, or at least installable via the install_github() R function?*

(1.) Using install_github() would make sense if the pipeline was written as an R-package itself, however this is not the case. The pipeline in intended to be run using a one-liner in a terminal command line (e.g. it’s a command line tool), the user will never directly open R, and as such the install_github() might not an obvious choice for installation. However, we do agree that the structuring of code for R-package management and installation could be improved upon, and we have therefore restructured it to no longer be a stand-along script. We have also modified the code to better handle the issue of different CRAN mirrors.

(2.)

- *the code does not appear to be under active development and I can’t find much evidence of userbase.*

The repository with the code is available at this link <https://github.com/ELELAB/CAncer-bioMarker-Prediction-Pipeline-CAMPP> where 95 commits are reported so far and the first author of this work is working on maintaining the code. With regards to the users, we recently open the link as public so we would expect more interest and activity in the next future and probably when the publication will be released. At the moment, we had some users testing it from other research groups but they wrote us issues in emails. In light of this we will encourage the practice of reporting the issues in the Github repository.

(3.)

- *it seems like the package itself provides a very small amount of new functionality, but I would defer to Mihaela’s opinion on this because I don’t work in cancer or biomarkers so I don’t know the state of the art. This assessment is purely based on my scan of the code.*

Although it does not include any novel bioinformatics approaches, it does entail a complete pipeline of analysis. The pipeline does everything from missing values imputation, clustering, variable selection (more than one method), co-expression analysis, survival analysis and interaction networks in one go, and results are automatically visualised with plots and tables. In our literature search for comparable tools we found very few tools which provided more than one, or a couple, of these features at once. As such the strength of CAMPP is not in its novelty, but in its complete framework and speed for the user.
